# Experimental evaluation of *Peromyscus leucopus* as a reservoir host of the *Ehrlichia muris*-like agent

**DOI:** 10.1186/s13071-017-1980-4

**Published:** 2017-01-28

**Authors:** Geoffrey E. Lynn, Jonathan D. Oliver, Ingrid Cornax, M. Gerard O’Sullivan, Ulrike G. Munderloh

**Affiliations:** 1 Entomology Department, University of Minnesota - Twin Cities, 1980 Folwell Ave, St. Paul, MN USA; 2 Masonic Cancer Center Comparative Pathology Shared Resource, Masonic Cancer Center, University of Minnesota - Twin Cities, 420 Delaware St. SE, Minneapolis, MN USA

**Keywords:** *Ehrlichia muris*, EMLA, *Ixodes scapularis*, Tick phenology, *Peromyscus leucopus*, Reservoir competence, *Anaplasmataceae*

## Abstract

**Background:**

The *Ehrlichia muris*-like agent (EMLA) is a newly recognized human pathogen in the North Central United States. Although blacklegged ticks (*Ixodes scapularis*) have been identified as capable vectors, wild reservoirs have not yet been established for EMLA. As key hosts for *I. scapularis*, white-footed mice (*Peromyscus leucopus*) are important reservoirs for various tick-borne pathogens, and potentially, for EMLA. The objective of this study was to evaluate reservoir competence in *P. leucopus* using a natural vector.

**Results:**

Mice acquired EMLA infection from feeding ticks and were able to transmit infection to naïve ticks. Transmission between simultaneously feeding tick life stages was also demonstrated. Infections in mice were acute and severe, with systemic dissemination. Limited host survival and clearance of infection among survivors resulted in a narrow interval where EMLA could be acquired by feeding ticks.

**Conclusions:**

*Peromyscus leucopus* is a competent reservoir of EMLA and likely to play a role in its enzootic transmission cycle. The duration and severity of EMLA infection in these hosts suggests that tick phenology is a critical factor determining the geographic distribution of EMLA in North America.

## Background

*Ehrlichia* is a genus of gram-negative intracellular bacteria transmitted by hard-bodied ticks. Several species are important medical and veterinary pathogens, including the *Ehrlichia muris*-like agent (EMLA), which has recently been recognized as a cause of human ehrlichiosis in Minnesota and Wisconsin [1, 2]. Surveillance and experimental studies indicate that the blacklegged tick (*Ixodes scapularis*) is the primary vector for EMLA [1, 3–9]. Despite the broad-ranging distribution of this vector throughout parts of North America, evaluation of over 75,000 blood samples collected between 2007 and 2013 from patients from all 50 USA states did not find evidence of human exposure outside of either Minnesota or Wisconsin [2]. Similarly, Stromdahl et al. reported finding no evidence of EMLA DNA in > 2000 *I. scapularis* collected at various military installations outside of these two states [9].

In contrast to the research associating *I. scapularis* with transmission of EMLA, the enzootic reservoirs of this pathogen are not yet well defined. *Borrelia burgdorferi* and *Anaplasma phagocytophilum*, the respective agents of Lyme disease and human anaplasmosis, the two most common tick-borne pathogens in the upper Midwest, are pathogens that also circulate in an enzootic transmission cycle between vertebrate hosts and *I. scapularis*. Although these ticks are generalists that feed on a variety of hosts including mammals, birds and reptiles [10, 11], the white-footed mouse (*Peromyscus leucopus*) is commonly recognized as a primary host, and a reservoir for pathogens transmitted by *I. scapularis*.

White-footed mice (WFM) are among the most ubiquitous, and frequently, the most numerous small mammals represented in surveys of northern deciduous forests [12–17]. As key hosts of immature *I. scapularis*, WFM permit heavy infestation and support a high level of molting success [18, 19]. These attributes and the demonstrated high reservoir competence of WFM for both *B. burgdorferi* [12, 20] and *A. phagocytophilum* strains responsible for human disease [14, 16, 21–23] frequently result in efficient production of infected nymphs, or high reservoir potential [24]. It is therefore possible that these mice could also be important reservoirs for EMLA. However, a survey of small mammals at two locations where EMLA is present in ticks found only 2/139 *P. leucopus* with DNA evidence of infection [3]. White-tailed deer (*Odocoileus virginianus*), the primary reservoir for *Ehrlichia chaffeensis*, another human pathogen closely related to EMLA, have not been found to carry EMLA in these same locations [3], even though deer are an important host for *I. scapularis*.

The primary goals of this study were to (i) assess whether WFM are susceptible to EMLA infection via infected nymph feeding, (ii) determine whether WFM are infectious for feeding larvae and for how long, and (iii) characterize pathogenesis in WFM infected with EMLA via tick bite.

## Methods

Ten female *P. leucopus* were sourced from the *Peromyscus* Genetic Stock Center at the University of South Carolina, (Columbia, SC, USA). Mice were approximately 16 weeks old at the time of study initiation. All research involving animals was performed in accordance with the recommendations in the Guide for the Care and Use of Laboratory Animals of the National Institutes of Health [25] and a protocol approved by The Institutional Animal Care and Use Committee (IACUC) of the University of Minnesota (# 1307–30753A).

Specific pathogen-free larval ticks used in the experiments hatched from eggs laid by gravid female *I. scapularis* that were obtained from Oklahoma State University. EMLA-infected nymphs were produced via larval feeding on hamsters inoculated with the EmCRT isolate cultured in ISE6 cells [7, 26, 27]. Successful hamster infection with EMLA was confirmed by testing blood using PCR with PER1 and PER2 primers complimentary to EMLA 16S rDNA under conditions described previously [7, 28]. DNA was extracted from blood using the Puregene Core Kit A (Qiagen Sciences, Valencia, CA, USA). Engorged ticks were washed and housed in 5-ml vented polystyrene tubes as described previously [7, 27]. At approximately 12 weeks following nymphal molt, 10–12 nymphs from a cohort in which 70% (*n* = 10) had tested PCR-positive for EMLA using the same primers as for testing blood, were placed on each mouse for transmission feeding. Ticks tested by PCR were surface sterilized with sodium hypochlorite, rinsed in purified water [7], and placed in sterile microfuge tubes where they were perforated with sterile needles prior to DNA extraction using the DNeasy Blood & Tissue Kit (Qiagen, Venlo, Limburg, Germany). PCR was performed using PER1 and PER2 primers with ~100 ng of template DNA. About 150–200 larvae were placed on individual *P. leucopus* for xenodiagnosis at varying time points between 24 h and 34 days following nymphal infestation.

Mice were screened for bacteremia as early as 24 h after nymph infestation. Blood for PCR was collected from the facial vein (live subjects), or by cardiac puncture following euthanasia. DNA was extracted from blood using the same method we described previously for hamsters. Following euthanasia, mouse tissues were removed aseptically and small sections were frozen at −70 °C, with the remainder fixed in 10% buffered formalin solution at 4 °C for 24–48 h, followed by storage in 70% ethanol. DNA was extracted from 10 to 20 mg of frozen tissue from each of the lung, heart, liver, kidney, spleen, and brain using the DNeasy Blood & Tissue Kit (Qiagen).

Fixed tissues were paraffin-embedded, sectioned at 4 μm onto slides (Fisher Scientific, Hampton, NH, USA), and a subset stained with hematoxylin and eosin (H&E) at the Masonic Cancer Center Comparative Pathology Laboratory at the University of Minnesota. Unstained paraffin-embedded tissue sections were processed using in situ hybridization (ISH), as previously described [7].

Histological images were acquired using a SPOT Insight 4.0 Megapixel color mosaic camera using Spot version 5.2 software. Images showing ISH were obtained using the SPOT equipment/software as well as a Nikon Color DS-Fi2 CCD camera and NIS Elements imaging software. Contrast, brightness, and sharpness were adjusted linearly across the whole images in Adobe Photoshop.

## Results

**Fig. 1:**
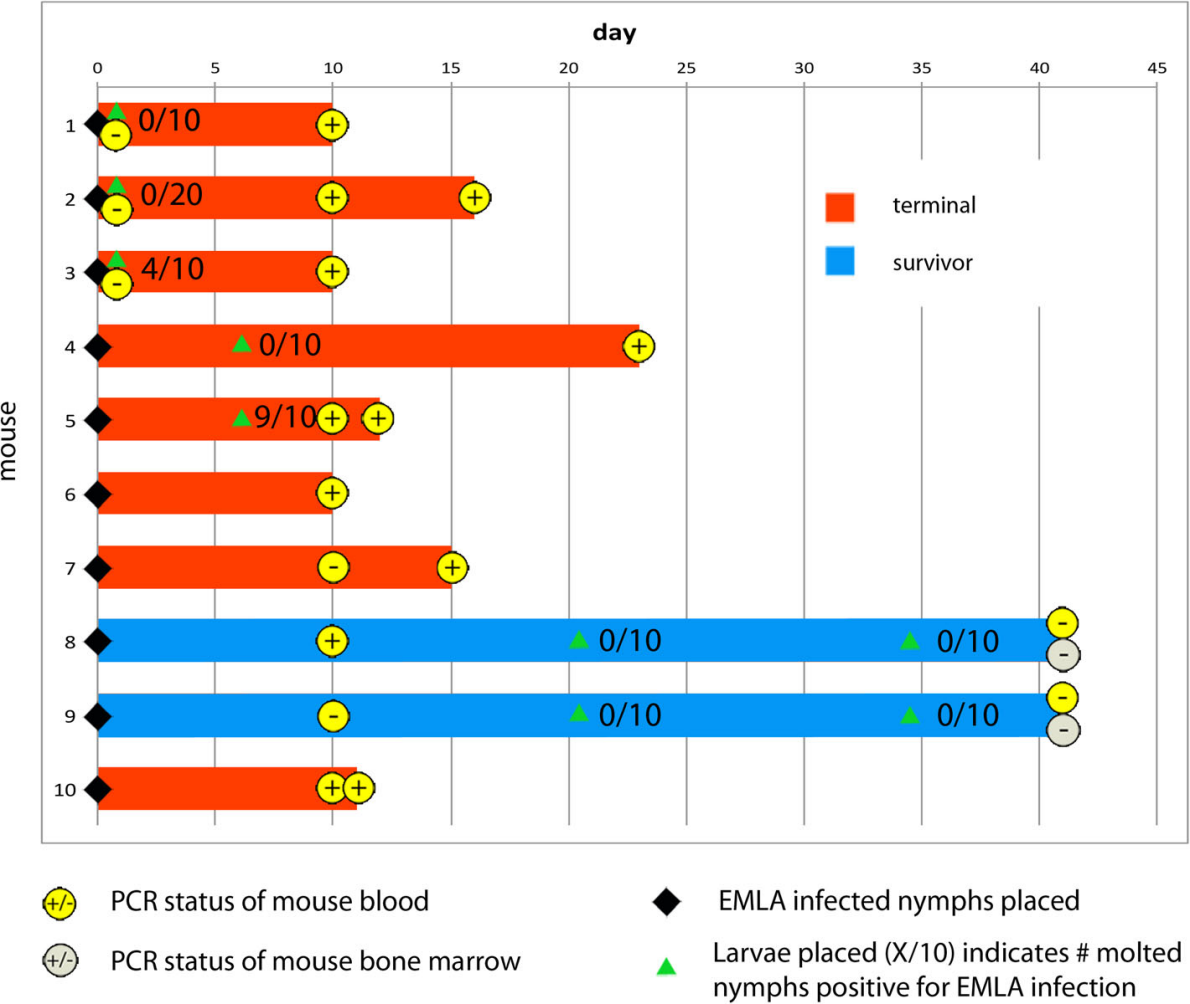
Timeline showing mouse survival and infectiousness for ticks in white-footed mice infected with EMLA via tick transmission. Infected nymphs were introduced on Day 0 followed by uninfected larvae at various time points. Tick and mouse infection status was determined by PCR

Nine of the 10 mice acquired EMLA infection from feeding nymphs, as evidenced by positive PCR results for blood (Fig. 1). Eight mice developed fatal disease and were subsequently euthanized. For the majority of individuals (5/8), this occurred within 10 to 12 days after nymph feeding was initiated. Onset of illness was sudden, with ataxia, tremors, and lethargy being the primary clinical signs, while dyspnea was not observed. Of the two mice that did not develop severe illness (#8 and #9), PCR evidence of infection was confirmed for one (#8) on day 10, while the second (#9) was negative by PCR at that time. It is possible that this second individual developed bacteremia after blood was collected on day 10. This sequence occurred for individual #7, which tested negative on day 10, but was subsequently positive when it developed severe illness on day 15.

Blood from three mice infested with larvae 24 h after infected nymphs were introduced tested PCR-negative for ehrlichiae at that time (Fig. 1). From those infestations, one mouse produced infected nymphs (4/10), while nymphs feeding as larvae on the other two mice tested negative. PCR results subsequently showed that all three mice had detectable EMLA bacteremia on day 10. Nine of 10 nymphs molting from larvae placed on mouse #5 seven days after infestation with infected nymphs tested positive. Because blood samples collected for mouse #4 on day 10 were inadequate for PCR, xenodiagnosis was utilized. Replete larvae that had dropped off mouse #4 from days 9–12 tested negative (0/10), as did newly molted nymphs (0/10) from this same larval feed. Mouse infection with EMLA was subsequently confirmed by PCR at the onset of illness, 23 days following infestation. Engorged nymphs were recovered from mouse #4 over the same time period as the ticks feeding on mouse #5, indicating that no delay of initiation of feeding by larval ticks had occurred, and that a prolonged incubation period resulted in the delayed onset of illness for this mouse. Each of the ticks tested (*n* = 40) that had fed on the two apparently healthy mice 21 and 34 days following nymphal infestation were negative.

**Fig. 2:**
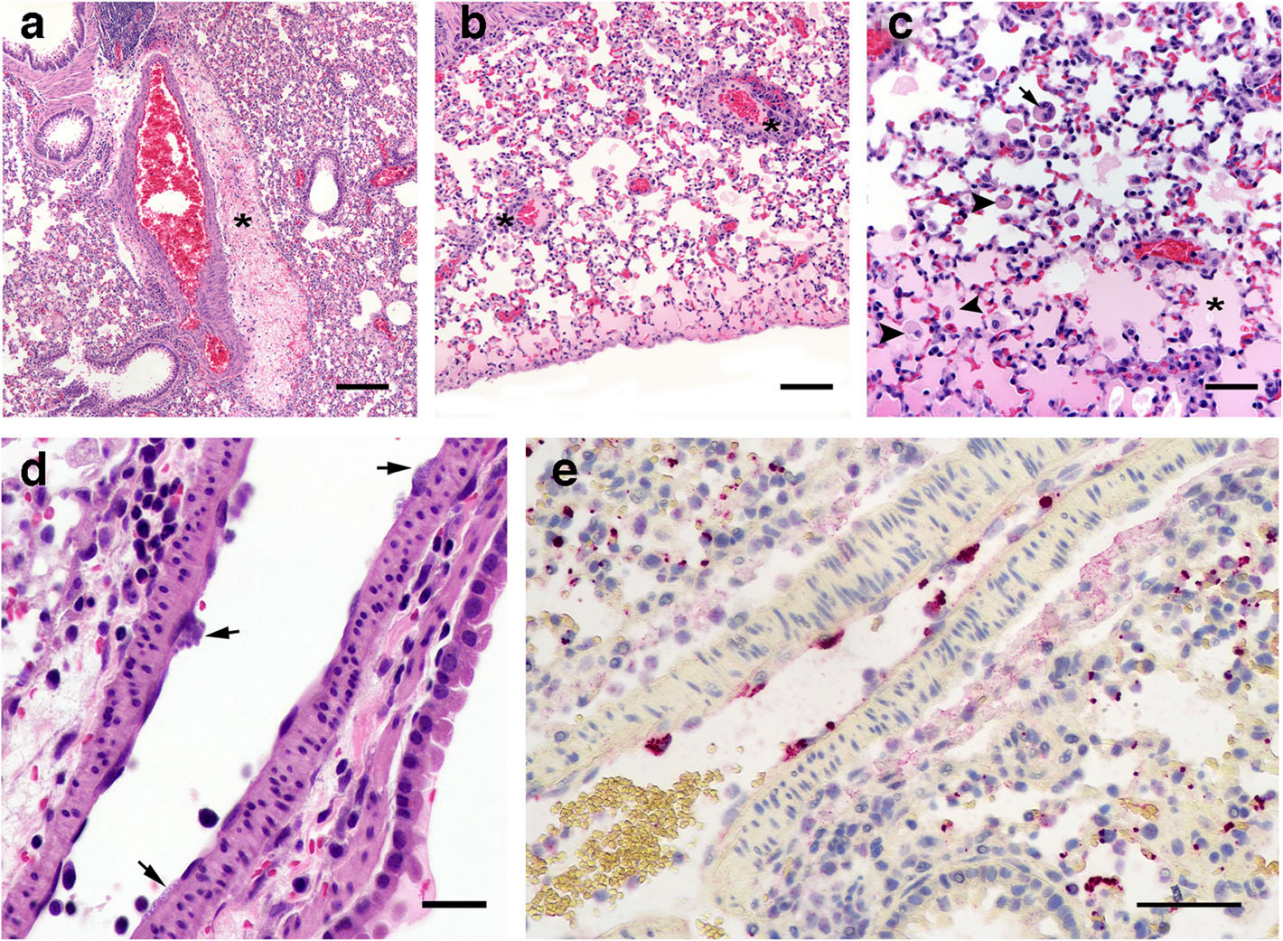
Lung tissue from white-footed mice acutely infected with EMLA. Images **a**–**d** show sections stained with hematoxylin and eosin, revealing various pathological signs including (**a**) marked perivascular edema (*asterisk*); (**b**) mononuclear perivascular infiltration in lung tissue (*asterisk*) and peripheral alveolar edema; (**c**) alveolar edema (*asterisk*), alveolar macrophages (*arrowheads*) and multinucleate macrophage (*arrow*); (**d**) enlarged vascular endothelial cells containing morulae (*arrows*). In situ hybridization was performed on section (**e**) to identify morulae within endothelial cells of pulmonary vasculature. EMLA is labeled *red*, cell nuclei are stained *blue. Scale-bars*: **a**, **b**, 200 μm; **c**, **e**, 100 μm; **d**, 50 μm

**Fig. 3:**
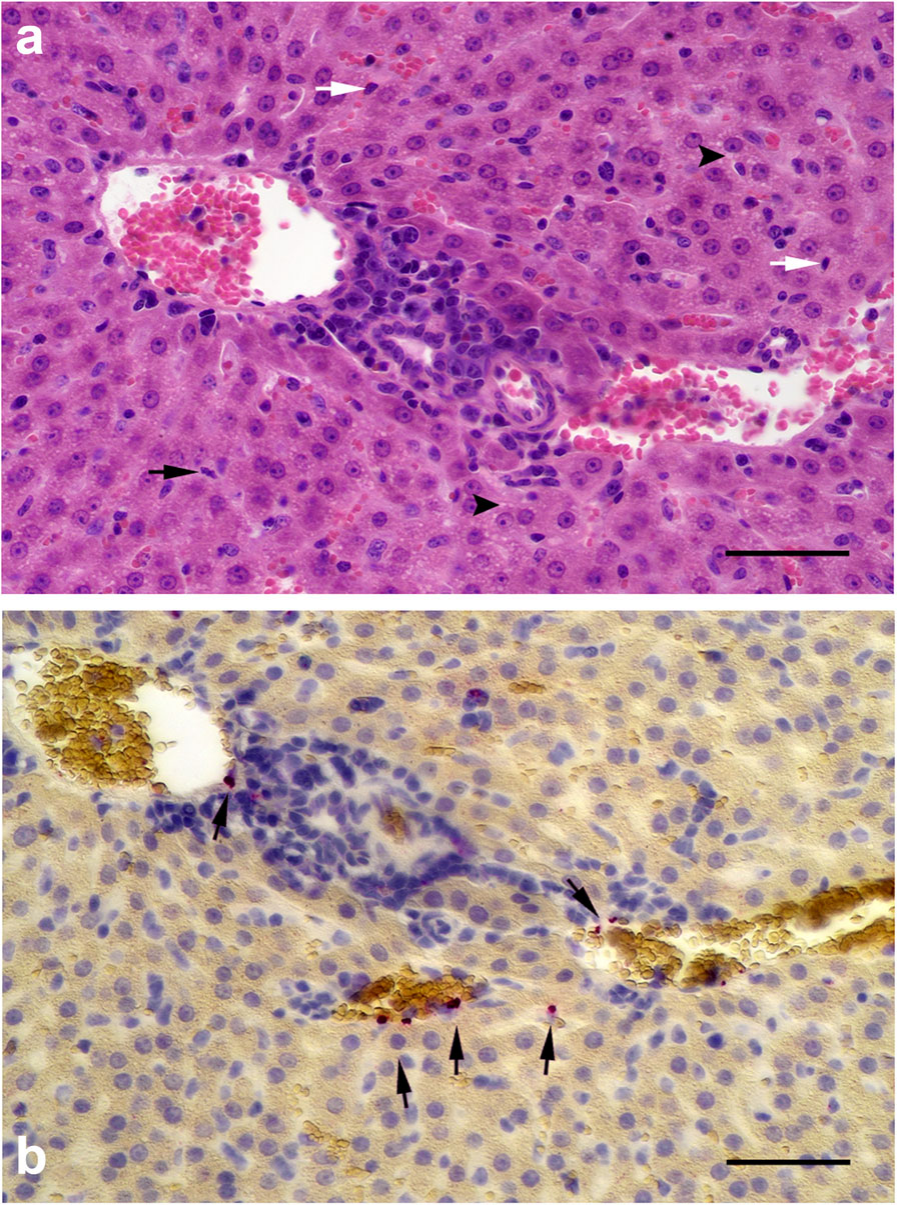
White-footed mouse liver tissue infected with EMLA. **a** Hematoxylin and eosin staining reveals lesions and other signs characteristic of inflammation, including a hepatic portal triad that is mildly infiltrated with mononuclear inflammatory cells. Kupffer cells in hepatic sinusoids are indicated by *white arrows*, and dividing Kupffer cells are designated by *black arrows*. The arrowheads point to small clear cytoplasmic vacuoles. **b** In situ hybridization labeling of morulae within hepatic or endothelial cells (*arrows*). EMLA is labeled red, cell nuclei are stained *blue. Scale-bars*: 100 μm

**Fig. 4:**
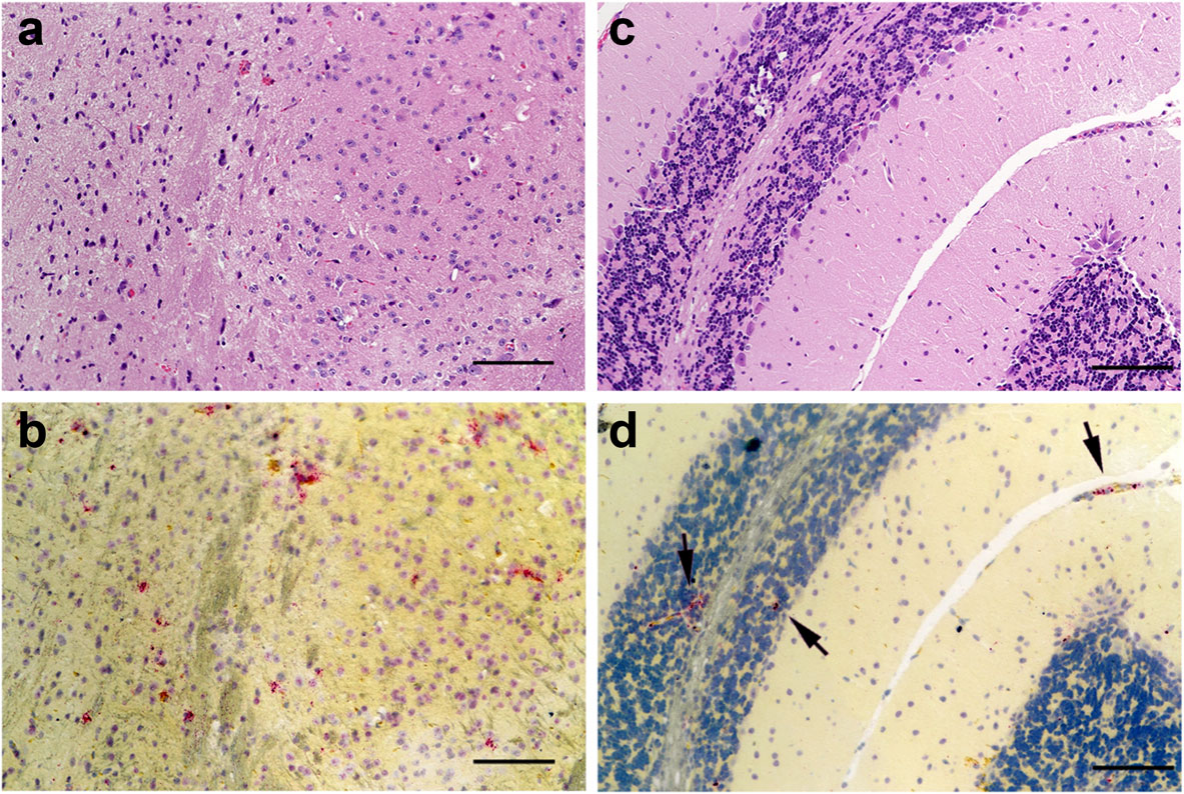
Brain tissue infected with EMLA. Images **a** and **b** display sectioned brain stem, while **c** and **d** show part of the cerebellum. Brain tissues lack overt signs of pathology as indicated by hematoxylin and eosin staining (**a**, **c**), despite the presence of EMLA in these same tissues as demonstrated by in situ hybridization where EMLA is labeled *red*, cell nuclei are stained *blue* (**b**, **d**). *Arrows* in **d** point to morulae. *Scale-bars*: 100 μm

All tissues (lung, heart, liver, kidney, spleen, and brain) obtained from the eight individuals with severe infections (mice #1–7, & #10) tested positive for ehrlichiae by PCR. These results were later confirmed for a combination of tissue sections from mouse #1 and mouse #2 using ISH (Figs. 2, 3 and 4). The two remaining mice (#8 & #9) were euthanized on day 41, when cardiac blood and all tissues including bone marrow tested negative by PCR.

**Fig. 5:**
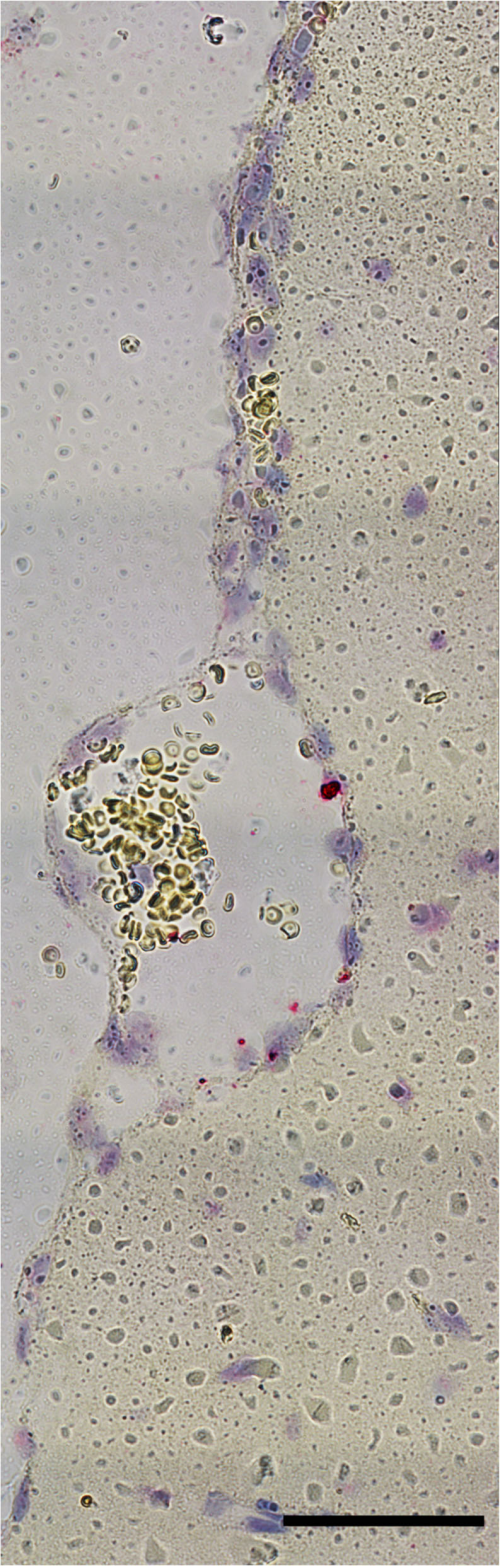
EMLA-infected vasculature within the leptomeninges, demonstrated using in situ hybridization. EMLA is labeled *red*, cell nuclei are stained *blue. Yellow* discs are erythrocytes. *Scale-bar*: 100 μm

Three of the mice that developed acute disease were examined by histology. The most significant lesion observed in all three mice was moderate interstitial pneumonia (pneumonitis) (Fig. 2a) characterized by multifocal thickening of the alveolar walls due to increased numbers of mononuclear inflammatory cells (Fig. 2b, c). In addition, there was marked diffuse perivascular edema (Fig. 2a), and moderate to marked peripheral alveolar edema with increased numbers of alveolar macrophages and occasional multinucleate cells (Fig. 2c). Pulmonary blood vessels were often surrounded by prominent lymphohistiocytic perivascular cuffs and contained large numbers of marginated intraluminal monocytes. Scattered endothelial cells were markedly expanded by vacuoles filled with a myriad of punctate organisms (Fig. 2d). The livers exhibited diffuse mild microvesicular hepatocellular vacuolation, moderate Kupffer cell hyperplasia, and increased numbers of periportal and sinusoidal mononuclear inflammatory cells (Fig. 3a). Examination of the spleens revealed moderate to marked expansion and disruption of the white pulp, frequent single cell lymphoid necrosis, increased numbers of tingible body macrophages, and infiltrates of large lymphoid cells and plasma cells extending into the red pulp. Similar disruption of lymphoid follicular architecture was noted in the mesenteric lymph nodes. Though brain tissues of acutely ill mice were extensively infected (Figs. 4 and 5), there were no obvious lesions present. A fourth mouse (#8) that tested positive by PCR but never showed overt signs of disease was also examined histologically, and no significant lesions were observed.

## Discussion

As both important hosts for immature *I. scapularis* and amplifying reservoirs of disease agents, WFM are likely to be important contributors to the natural enzootic cycle of EMLA. Here we show that EMLA-infected *I. scapularis* nymphs are capable of infecting WFM through blood-feeding. Moreover, uninfected larvae feeding simultaneously with infected nymphs can acquire infection shortly after a host is exposed to EMLA. While previous research using inbred laboratory mice demonstrated that bacteremia was absent or very low during the first week following intraperitoneal (i.p.) infection, peaking at about 2 weeks [8], we observed successful acquisition by larvae feeding within a period of 24 h to 5 days after infestation with infected nymphs. Co-feeding transmission of EMLA by *I. scapularis* feeding on C57BL/6 mice (*Mus musculus*) has been reported [8] and is also likely to occur with the related *A. phagocytophilum*, where tick-to-mouse transmission can occur within 24 h and ticks may acquire the pathogen even when host bacteremia is too low to detect by PCR [23, 29]. The high rate of acquisition (90%) of EMLA by ticks fed on an acutely ill individual with detectable bacteremia (#5) we observed was comparable to that reported for larvae fed on needle-infected C57BL/6 mice (90–100%) at peak bacteremia [5]. Ticks feeding during the same time period on a second mouse (#4) that became severely ill much later did not acquire EMLA. While PCR was not performed for this individual at the time of infestation, onset of illness was much later (23 days) than in the other mice, which may be attributable to the phenotypic variability previously observed in these outbred mice in response to infection [30]. Although the quantity of infected nymphs used to infest mice was consistent, it is also possible that differences in bacterial inoculum due to host grooming or tick bacterial load could account for this outlier.

Similar to inbred laboratory mice, tick transmission of EMLA resulted in systemic dissemination of ehrlichiae to various organs of WFM [5, 8]. The frequency of severe outcomes we observed (80%) falls within the range of those previously reported for C57BL/6 mice following tick transmission (27% and 80%) [5, 8]. Neither xenodiagnosis nor PCR of bone marrow demonstrated chronic EMLA infection in our WFM infected by ticks, in contrast to previous reports using different experimental conditions to infect inbred laboratory mice [31].

Histological analysis of WFM indicated that the high rate of morbidity was a result of severe, acute infection that caused multifactorial disease. Lesions were observed in multiple organs, most prominently in the lungs, liver, and lymph nodes. As reported for the IOE mouse model of ehrlichiosis, the rapid course of disease in WFM precluded the formation of liver granulomas in WFM [32]. Pulmonary edema, though present, was limited compared with our previous observations in i.p. inoculated C57BL/6 mice (unpublished data). Varied presence of EMLA in all tissues assayed was demonstrated by ISH and confirmed by PCR results. EMLA has previously been cultured in human and monkey endothelial cells as well as in an *I. scapularis* cell line predominantly comprised of neuron-like cells [7, 26]. *Anaplasma phagocytophilum* and *E. chaffeensis* reside in leukocytes, which allows for their dispersal to various organs, and it is possible that endothelial cells of the vasculature and lymphatic systems facilitate systemic dissemination of EMLA by releasing bacteria into the blood or lymph. Of particular interest in our study was the extensive presence of EMLA in vascular endothelial cells throughout the brain. Yet despite the clinical signs we observed suggesting possible neurological disease, pathological lesions were not obvious in these mice. There was, however, some evidence of mild cerebral edema, which should be further investigated.

Our results establish WFM as competent reservoirs of EMLA, although the reservoir potential of this species appears to be limited by a short duration of infectiousness in the hosts, reducing potential acquisition opportunities for larvae. This is in contrast to the primary pathogens of medical importance transmitted by *I. scapularis*, *Borrelia* spp. and *A. phagocytophilum*, which do not typically induce clinical disease or affect the survival of WFM and other wild rodent hosts [33–40]. Rather, they cause extended infections that can persist for months to years [14, 20, 36, 38, 40–50], allowing much greater opportunity for transmission to naïve vectors. Acute, lethal EMLA infections in WFM, a host that typically accounts for a large proportion of larval bloodmeals in the midwestern and northeastern USA are likely to limit presence of the pathogen in nature, which corresponds with available surveillance data. Nonetheless, it cannot be ruled out that EMLA infections may be less severe and longer lasting in other potential reservoir species that might consequently play a greater role in its natural maintenance.

In addition, factors including highly efficient acquisition by larvae, and tick phenology in areas of EMLA endemicity may counteract the limitations of a brief period of host infectiousness. Most bacterial pathogens transmitted by *Ixodes* ticks are transstadially retained, but not passed vertically from females to offspring (transovarial transmission). Consequently, the seasonal activity of tick life stages relative to each other may have considerable impact on transmission of certain pathogens that do not persist in hosts for long periods. Specifically, successful maintenance of these pathogens in nature may require that larval feeding occurs in close enough temporal proximity to infected nymphs (or potentially adults) for hosts to be infectious for ticks.

As such, previous studies have demonstrated that vector phenology influences distribution patterns of tick-borne encephalitis virus (TBEV), *B. burgdorferi* and *A. phagocytophilum* [51, 52]. TBEV infections are brief and non-systemic in rodent hosts, requiring simultaneous feeding of infected nymphal and larval *Ixodes ricinus* ticks for transmission to naïve ticks to occur, and as a result, geographic distribution of the virus is limited to areas with a high degree of seasonal synchrony of immature stages of the vector [53]. In contrast, *A. phagocytophilum*, which often infects hosts for weeks or months, is present in the USA both in areas where immature *I. scapularis* activity is synchronized, and those where activity is asynchronous. Furthermore, local genotypic composition of *B. burgdorferi* populations has been shown to be influenced by phenology, where strains that infect hosts for longer periods are dominant in areas where feeding of larvae and nymphs is asynchronous [54].

Likewise, phenology of tick life stages would appear to impact distribution of EMLA. In the Northeastern US, feeding patterns of immature stages of *I. scapularis* are typically more asynchronous than in the Upper Midwest [54], with nymphal peaks in late spring and early summer, followed by larval peaks toward the latter part of summer into early fall [55–58]. In contrast, peak feeding periods of immature *I. scapularis* life stages in the Upper Midwest are much more closely aligned, with extensive overlap in some locations [16, 17, 54] that is more conducive for inter-stage transmission of pathogens that cause short-duration infections in hosts. The results of this study support the previous conclusions of Karpathy et al. [8], that the current geographical distribution of EMLA is likely a consequence of these disparate phenological patterns [17, 54]. However, the broad spatial and temporal continuum of phenology occurring in *I. scapularis* populations between the Northeastern and North Central USA would suggest that additional factors contribute to the relatively limited geographical presence of EMLA as currently observed.

Our findings suggest that the transmission dynamics of EMLA appears to lie somewhere between that of TBEv and *B. burgdorferi*, where tick acquisition of ehrlichiae occurs both during a short period of bacteremia as well as potentially during co-feeding transmission from infected nymphs to larvae. The latter would extend the period during which ticks could become infected by several days prior to onset of bacteremia. It is also possible that co-feeding transmission enables species that otherwise might not be considered important reservoirs given their inability to develop systemic infection, to contribute to enzootic maintenance of EMLA [53]. Further studies should investigate the possibility that wild canids, insectivores, and other vertebrates, in addition to rodents, play a role in the enzootic cycle of EMLA, with sampling efforts conducted at or shortly after the expected peak nymphal feeding period.

## Conclusions

Nymphal *I. scapularis* feeding on *P. leucopus* successfully transmitted EMLA to their hosts, whereupon naïve larval *I. scapularis* were then able to acquire EMLA from these same hosts for a short period following nymphal feeding. The majority of mice in this study developed severe disease. Ehrlichiae were widely disseminated in the hosts, particularly in vascular endothelial cells, and pathological findings were especially prominent in the lungs, liver and lymphoid tissues. Although *P. leucopus* were determined to be competent reservoirs for EMLA, the brief period of infectivity and high mortality observed in this species indicates that (i) local patterns of *I. scapularis* phenology may be critical in determining the geographical distribution of the pathogen, and (ii) there are likely to be other important host species for EMLA.

## Abbreviations

EMLA: *Ehrlichia muris*-like agent
TBEV: Tick-borne encephalitis virus
WFM: White-footed mice
